# *Arhgap29* deficiency causes EEC like syndrome in mice

**DOI:** 10.1016/j.gendis.2024.101404

**Published:** 2024-09-07

**Authors:** Dandan Chi, Yumeng Wang, Lili Yu, Wenyan Ruan, Beibei Zhang, Jian Ma, Xiaohong Duan, Yongqing Huang

**Affiliations:** a Department of Oral and Maxillofacial Surgery, Hospital of Stomatology, The General Hospital of Ningxia Medical University, Yinchuan, Ningxia 750004, China; b Ningxia Medical University, Yinchuan, Ningxia 750004, China; c Ningxia Key Laboratory of Oral Disease Research, Ningxia 750004, China; d Ningxia Key Laboratory of Craniomaxillofacial Deformities Research, Ningxia 750004, China; e State Key Laboratory of Oral & Maxillofacial Reconstruction and Regeneration, National Clinical Research Center for Oral Disease, Shaanxi Key Laboratory of Stomatology, Department of Oral Biology & Clinic of Oral Rare Diseases and Genetic Diseases, School of Stomatology, The Fourth Military Medical University, Xi'an, Shaanxi 710032, China

Cleft lip/palate (CL/P) is generally divided into two main types, non-syndromic and syndromic CL/P, with a global incidence of approximately 1 in 700.^1^ Previous studies have identified various susceptibility genes associated with non-syndromic CL/P, while the pathogenic gene of syndromic CL/P and the related mechanisms are not well documented. Ectrodactyly–ectodermal dysplasia–cleft lip/palate (EEC) syndrome is a representative syndromic CL/P, which is characterized by mild-to-severe symptoms such as missing or irregular fingers or toes, cleft lip or palate, distinctive facial features, and abnormalities of the eyes and urinary tract. The mutations in *TP63* have been associated with some EEC cases. Meanwhile, there were other reported EEC cases without mutations in *TP63* or other certain genes, suggesting other potential pathogenic genes involved in EEC syndrome. *ARHGAP29* encodes Rho GTPase-activating protein 29, broadly expressed in various tissues such as blood, liver, and heart, especially in the palatal shelf.^2^, ^3^, ^4^ *Arhgap29* mutant mice presented abnormal oral epithelial adhesions,^5^ *ARHGAP29* has also been regarded as the susceptible gene of non-syndromic CL/P since 2012. Our whole exome sequencing also identified a new missense mutation(c.451C>T, p.Leu151Phe) in *ARHGAP29* in a Chinese non-syndromic CL/P pedigree (Fig. S2C).

However, the close relationship between *ARHGAP29* and CL/P and the involved molecular mechanism were not clear. To explore in-depth the phenotypes and mechanisms whereby *ARHGAP29* deficiency, here we established *Arhgap29* knockout mice with CRISPR/Cas9 technique and found the mice presenting EEC-like phenotypes, revealing a new role of *Arhgap29* leading to syndromic CL/P.

The deletion of *Arhgap29* reduced the ARHGAP29 protein level in various tissues (Fig. S1B, C) and most knockout mice could not survive more than postnatal seven days (P7). The newborn *Arhgap29*^*−/−*^ mice (P0) were significantly smaller than wild-type littermates and had abnormal oral fissures (Fig. 1A). *Arhgap29*^*−/−*^ mice at embryonic day 14.5 (E14.5) exhibited a different degree of ocular abnormality, including slight microphthalmia with a slightly abnormal pupil shape, moderate microphthalmia, and severe microphthalmia (Fig. 1A; Fig. S3). E14.5 *Arhgap29*^*−/−*^ mice also showed ectrodactyly in the forefoot and kinked tail tips (Fig. 1A; Fig. S3). P0 *Arhgap29*^*−/−*^ mice exhibited a complete cleft palate (Fig. 1B) and disappeared palatal shelf using micro-CT scanning (Fig. 1B). During the palatogenesis, palatal shelves of the wild-type mice elevated to reach the midline, and medial edge epithelial presented at E14.5. *Arhgap29*^*+/−*^ embryos exhibited a delay in development and could not reach the midline of the palate from the anterior palate, medial, to the posterior palate, while *Arhgap29*^*−/−*^ mice had a more severe phenotype (Fig. 1C). At E15.5, the control palatal shelves displayed completed fusion and medial edge epithelial disappeared. Palatal shelves of *Arhgap29*^*−/−*^ mice had elevated; however, the anterior palate, medial, and posterior palate had a fusion failure of palatal shelves (Fig. S4). At E16.5, the control palatal shelves displayed a mature stage of palatal development, but *Arhgap29*^*−/−*^ mice showed a complete cleft palate from the anterior palate, medial, to the posterior palate (Fig. S5). These data indicated that the loss of *Arhgap29* gene hindered the growth and development of the palatal shelves at a critical stage.

**Figure. 1:**
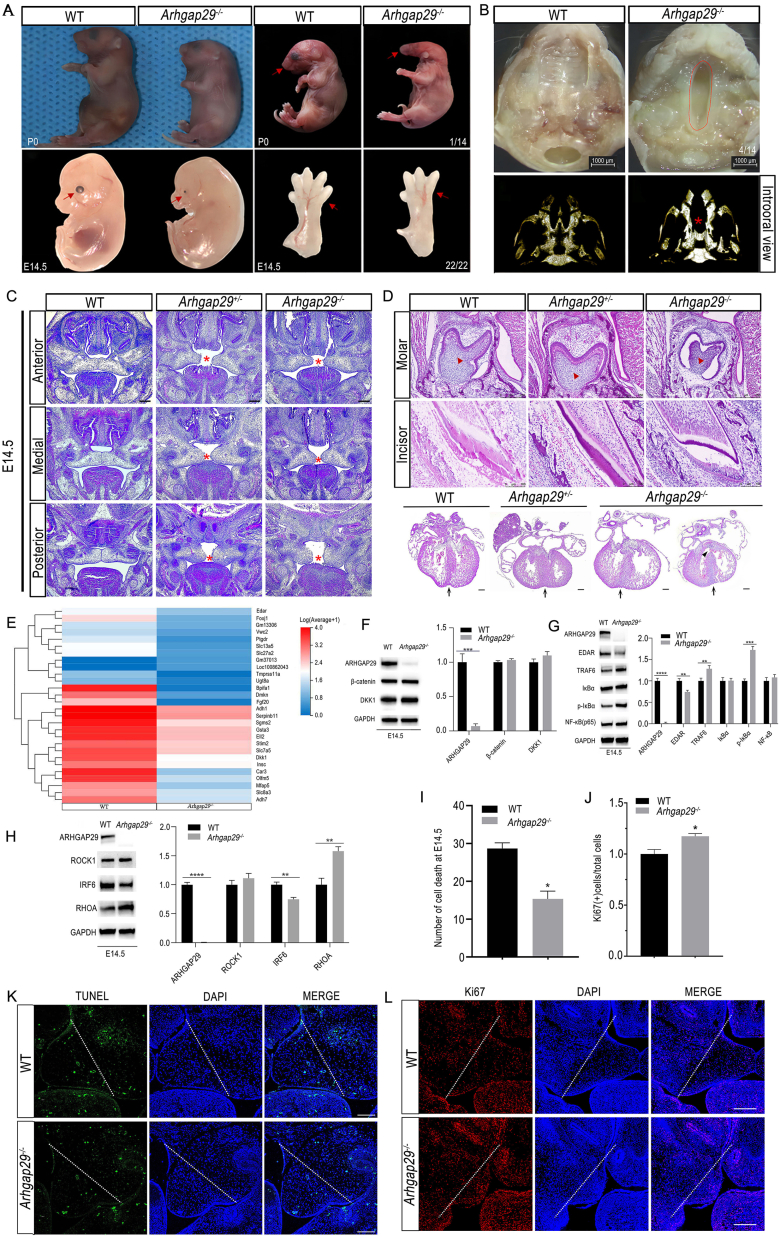
Phenotypes and the involved mechanisms in *ARHGAP29* deficiency mice. **(A)** General appearance of P0 and E14.5 mice of the wild-type and *Arhgap29*^*−/−*^ mice. Red arrow points tothe abnormal mouth, eye, or finger; the lower right digit represents penetrance of the phenotype. **(B)** Intraoral views and three-dimensional reconstructions from micro-CT tomography scans of the palate of control and *Arhgap29*^*−/−*^ (P0) mice. The red dotted line, cleft palate with 28.57% penetrance (4/14); red asterisk, palate shelf. **(C)** Palatal shelves images with hematoxylin-eosin staining. The palatal developmental defects in E14.5 *Arhgap29*^*−/−*^ and *Arhgap29*^*+/−*^ mice were compared with wild-type mice at anterior, medial, and posterior palate. Asterisk, palate shelf; scale bar, 500 μm. **(D)** Tooth and heart images with hematoxylin-eosin staining. The molar germs and incisor germs at P0 and heart at E14.5 displayed developmental defects in *Arhgap29*^*−/−*^ and *Arhgap29*^*+/−*^ mice compared with wild-type mice. Red arrow, tooth germ; arrow, ventricular septal defect; black arrows at the bottom, interventricular sulcus. **(E)** Heatmap of RNA-sequencing of control and *Arhgap29*^*−/−*^ palates at E14.5. Horizontal axis, a sample of control and *Arhgap29*^*−/−*^ mice; vertical axis, differential genes; red, vary widely; blue, vary lowly. **(F–H)** Western blots of wild-type and *Arhgap29*^*−/−*^ palates at E14.5 for (F) WNT signaling mediators, (G) NF-κB signaling mediators, and (H) IRF6/RHOA signaling mediators. Right histogram, quantification of western blots from the left panels. Data are representative of three independent samples for Western blot. Error bars indicate standard deviation (SD). ∗*p* < 0.05, *p*-values are calculated from the student's *t*-test. **(I)** The number of TUNEL-positive cells was counted on the left of the white dashed line. Error bars indicate SD. ∗*p* < 0.05. **(J)** The number of Ki67-positive cells was counted on the left of the white dashed line. Error bars indicate SD. ∗*p* < 0.05. **(K)** TUNEL staining of E14.5 control and *Arhgap29*^*−/−*^ palate shelves. **(L)** Immunofluorescent staining for Ki67 in the palatal shelves of wild-type and *Arhgap29*^*−/−*^ mice. Scale bar, 100 μm.

Next, we observed other phenotypes such as tooth and heart in *Arhgap29* deficient mice. P0 *Arhgap29*^*−/−*^ mice had malformed molar germs and incisor germs and enamel dysplasia compared with wild-type ones (Fig. 1D). In addition, *Arhgap29*^*−/−*^ mice presented pale body surface of hearts suggesting insufficient blood supply (Fig. S3). Further histological analysis found that both homozygous and heterozygous mice had abnormal heart shape. E14.5 *Arhgap29*^*−/−*^ embryos exhibited more severe cardiac defects such as rounded ventricular wall or ventricular septal defect and loosely arranged cardiomyocytes (Fig. 1D). No significant morphological abnormalities were found in other important organs of E14.5 *Arhgap29*^*−/−*^ embryos (Fig. S6A–F). The cardiac defects might explain the perinatal deaths of most *Arhgap29*^*−/−*^ mice.

Then we performed RNA-sequencing analysis of palatal shelves in wild-type and *Arhgap29*^*−/−*^ embryos at E14.5 to screen out the possible downstream molecular pathways related to *Arhgap29* deletion. The expression of 33 genes showed significant changes compared with the control littermates (*p* < 0.05) (Fig. 1E). KEGG database analysis enriched the changes to Wnt and NF-κB (nuclear factor kappa B) pathways, both of which are critical for embryonic development. Then we evaluated whether DKK1 (dickkopf1), EDAR (ectodysplasin A receptor), Wnt, and NF-κB signaling pathways were altered in palatal shelves of E14.5 *Arhgap29*^*−/−*^ embryos via Western blot. No significant difference was found in the expression of DKK1 and WNT signaling-associated proteins (Fig. 1F). The detection of NF-κB pathway showed the increased level of p-IκBα (phosphorylated inhibitor kappa B-alpha) and TRAF6 (TNF receptor associated factor 6) and unchanged NF-κB (P65) in *Arhgap29*^*−/−*^ mice (Fig. 1G). These results suggest that the deletion of *Arhgap29* may affect palatogenesis through NF-κB pathway. As a reported pathogenic gene of tooth agenesis, the reduced level of EDAR could explain the abnormal changes of tooth germs in *Arhgap29*^*−/−*^ embryos.

Previous studies found that *Arhgap29* expression was decreased in all epithelia of *Irf6* (interferon regulatory factor 6) deficient embryos, leading to increased levels of active RhoA (Ras homolog family member A). It was surprising that IRF6 expression was reduced and ROCK1 and RHOA levels were increased in our *Arhgap29*^*−/−*^ mice (Fig. 1H).

Palatal growth and development are regulated by palatal mesenchymal cell proliferation and apoptosis. The NF-κB pathway regulates cell proliferation and survival and is involved in various life processes. To investigate whether these biological activities were changed in *Arhgap29*^*−/−*^ mice, we examined TUNEL-positive cells in the palatal mesenchyme in control and *Arhgap29*^*−/−*^ mice at E14.5 and found *Arhgap29*^*−/−*^ embryos had less TUNEL-positive cells (Fig. 1I–K). In addition, we performed immunofluorescent staining for proliferating cell nuclear antigen (Ki67), to investigate whether *Arhgap29*^*−/−*^ mice affected cell proliferation in the palatal mesenchyme. The results suggested that Ki67 was strongly expressed in palatal epithelial and mesenchymal tissues in *Arhgap29*^*−/−*^ compared with wild-type mice (Fig. 1J–L). These results indicated that *Arhgap29* deficiency promoted embryonic palatal mesenchymal cell (EPMC) proliferation and inhibited EPMC apoptosis.

TP63 locates in the upstream of IRF6, and its homologue ΔNp63α interacts with the NF-κB pathway in epithelial homeostasis. One plausible explanation is that *Arhgap29* deletion regulates NF-κB and IRF6 signaling to influence TP63 expression, potentially leading to EEC-like syndrome. In summary, we successfully established the *Arhgap29* deficiency mice model presenting EEC syndrome like phenotypes. Our research provides a new perspective on the pathogenesis of syndromic CL/P.

## Ethics approval

Approval for this study on human and animal experiments were gained from the Medical Research Ethics Review Committee of General Hospital of Ningxia Medical University (No. 2015-076, 2019-057). We declared the written informed consents were obtained from all participants in the manuscript.

## Author contributions

Dandan Chi, Yumeng Wang, and Lili Yu contributed to the conception and design, acquisition, analysis, and interpretation, drafted the manuscript, and critically revised the manuscript. Wenyan Ruan contributed to acquisition and analysis, and critically revised the manuscript. Beibei Zhang contributed to acquisition and critically revised the manuscript. Jian Ma contributed to the conception and design and critically revised the manuscript. Xiaohong Duan contributed to the conception and design, analysis, and interpretation, and critically revised manuscript. Yongqing Huang contributed to the conception, acquisition, and interpretation, and critically revised the manuscript. All authors gave their final approval and agreed to be accountable for all aspects of the work ensuring integrity and accuracy.

## Data availability

The authors declare that the data supporting the findings of this study are available within the article and its supplementary information files.

## Funding

This work was supported by grants from the 10.13039/501100001809National Natural Science Foundation of China (No. 81960197, 82370907, 81974145), the Key R&D Plan of Shaanxi Province, China (No. 2021ZDLSF02-13), the Key Project of National Clinical Research Center for Oral Diseases (China) (No. LCC202201), the Open Projects of General Hospital of Ningxia Medical University (No. 2019209), and the Comprehensive Reform of Public Hospital-Key Discipline Construction and Talent Training Project of General Hospital of Ningxia Medical University (No. NYDZY035).

## Conflict of interests

All authors declared no conflict of interests.
