# Supplementary material for: Arhgap29 deficiency causes EEC like syndrome in mice
Source: Genes Dis. 2024 Sep 7;12(4):101404. doi: 10.1016/j.gendis.2024.101404 (PMC11995055; doi:10.1016/j.gendis.2024.101404)
Supplement: Multimedia component 1 [file mmc1.docx]

**Supplementary Material**

**Materials and methods**

**Generation of the *Arhgap29* knockout mouse model**

To investigate the biological function of the *Arhgap29* gene in mice, we generated a novel *Arhgap29* gene knockout mouse model by CRISPR/Cas-mediated genome engineering. Cas9 and gRNA were co-injected into fertilized eggs to delete *Arhgap29* exons 4–12 (Fig. S1A; Cyagen Biosciences Inc.), which resulted in a frameshift mutation predictably and affected all transcripts. Homozygous and heterozygous mice were labeled as *Arhgap29^−/−^* and *Arhgap29^+/−^,* respectively. The experimental unit is a single mouse. All mice were group-housed under routine conditions in a 12-h day/night cycle with free availability of diet and water supplementing every two days. To determine the genotype of offspring, we normally cut off 2–3 mm tail tissue from each mouse for DNA extraction and PCR identification. The PCR primers are as follows: F1:5’-TTCAGGCAGAAGTAGAATCTGTTCA-3’,R1:5’-GTTAGTATGAGGCCAAGCAGAATCT-3’; F2:5’-TTCAGGCAGAAGTAGAATCTGTTCA-3’,R2:5’-TATTTCCTATCAACAGGCCCTTGG-3’. C57BL/6 female mice (8-10 week old and 20g weight) obtained from the experimental animal center of the Fourth Military Medical University were mated overnight, and a vaginal plug formation was defined as embryonic day (E0.5). The mice from the same litter were used for each comparison. We collected wild type mice (n=72), *Arhgap29^+/−^* (n=105) and *Arhgap29^−/−^* (n=50) for phenotype observation and histochemical staining, three independent samples for each genotype for RNA sequencing and western blot. All animal experiments complied with the protocols for animal use, treatment, and euthanasia approved by Medical Research Ethics Review Committee of Ningxia Medical University General Hospital (2015-076). And the animal research is reported according to the ARRIVE guidelines.

**Priori sample size calculation**

C57BL/6 mice virtually never has cleft palate under natural conditions (p_e_ = 0), 20% *Arhgap29^-/-^* mice were estimated that would occur cleft palate (p_c_ = 0.2). In this study, the desired statistical power was set to 0.8 (1-β = 0.8), the level of significance is 0.05 (α = 0.05) and the desired effect size is 0.8 (f = 0.8, C= 7.85), The estimate for the minimum sample size is computed from the formula below (n_min_ = 44). n=C(p_c_q_c_+p_e_q_e_)/d^2^+2/d+2, annotation:p = 1-p, d = |p_c_-p_e_|.

**Immunofluorescence staining procedures**

Embryos were collected at E14.5, E15.5, and E16.5 after parturition by cesarean operation and fixed in 4% paraformaldehyde. This step was followed by paraffin embedding and sectioning (4μm) for hematoxylin and eosin (H&E) and immunofluorescence staining. Anatomic markers, such as eyes and first molars, were used to ensure that the sections were taken from the same location as previously described^36^. After dewax and hydration, the paraffin sections, antigen retrieval was performed using 0.01 mol/L Citrate Antigen Retrieval Solution (pH 6.0) (G1202, Servicebio, Wuhan, China) in microwave oven (medium fire 8 minutes, stop fire 8 minutes and turn to medium low fire for 7 minutes). After natural cooling, they were washed with PBS (pH 7.4) 3 times (each time for 5 minutes) on a shaker. Then, the sections were blocked with 5% BSA for 30 minutes at room temperature. Thereafter, the sections were incubated with rabbit anti-mouse Ki67 antibody (GB151499, Servicebio, Wuhan, China) at 4°C overnight incubation. The sections were washed with PBS and incubated with fluorescent secondary antibody (Thermo Fisher Scientific, USA) in dark for 50min at room temperature. After washed with PBS 3 times, the sections were incubated with DAPI for 10 minutes in dark room temperature. Finally, the sections were washed with PBS 3 times, sealed with Fluorescence decay‐resistant Medium (G1401, Servicebio) and placed under a fluorescence microscope to observe and collect images.

**Micro-CT scanning and three-dimensional reconstruction**

Mouse heads were collected from wild-type and *Arhgap29^−/−^* newborn mice (P0) and fixed in 4% paraformaldehyde at 4 °C overnight. The heads were scanned by Micro-CT system setting to 80 keV, 500 mA. The three-dimensional reconstruction was performed with the Data Reviewer software.

**RNA extraction and RNA sequencing**

The embryos were harvested at E14.5, and heads were collected in coldly sterile PBS. After the lower jaws were removed under a dissecting microscope and the status of the palatal shelves was assessed, palatine tissues were carefully taken out using microscissors and microforceps. The tissues were quickly frozen in an ice box and stored at −80 °C for total RNA extraction by GoldHi Plasmid Mini Kit (CWBIO, China).

RNA sequencing was performed on the cDNA library of wild-type mice and *Arhgap29^−/−^* using the DNBSEQ platform (BGI, China). Three biological replications were contained in one group. The pheatmap function was used to draw a differential gene clustering heatmap. Functional classification and annotation for the biological process of differential expression genes were performed based on GO and KEGG databases.

**Western blot analysis**

Palatine tissues were collected from E14.5 mice, and the extraction process was described above. Palatal proteins of a mouse embryo were extracted with 100-µL RIPA lysis buffer, 1-µL phenylmethylsulfonyl fluoride, and 2-µL phosphatase inhibitor using a tissue homogenizer. Tissue samples were then placed on ice for 10 min, and the lysate was collected and centrifuged at 12,000 rpm for 15 min. Next, the lysate was diluted with 5×SDS-PAGE sample loading buffer and boiled at 95 °C for 5 min. Following that, protein was used for SDS-PAGE and transfer, and membranes were incubated with mouse anti-mouse ARHGAP29 antibody (Santa, USA), mouse anti-mouse IRF6 antibody (Santa, USA), mouse anti-mouse ROCK1 antibody (Santa, USA), mouse anti-mouse DKK1 antibody (Santa, USA), mouse anti-mouse β-catenin antibody (Santa, USA), mouse anti-mouse EDAR antibody (Santa, USA), rabbit anti-mouse RHOA antibody (Abcam, Britain), rabbit anti-mouse TRAF6 antibody (Abcam, Britain), mouse anti-mouse IκBα antibody (Cell Signaling Technology, USA), rabbit anti-mouse P-IκBα antibody (Cell Signaling Technology, USA), rabbit anti-mouse NF-κB antibody (Cell Signaling Technology, USA), rabbit anti-mouse GAPDH antibody (Zen-Bioscience, China) at 4 °C overnight. This step was followed by incubation with goat anti-rabbit/mouse IgG (Proteintech, China) at room temperature for 50 min, and images were detected using Western ECL Imager (VILBER FUSION FX, France).

**Analysis of apoptosis**

All sections were given at E14.5. Apoptosis assays were performed using a TUNEL staining kit (Bioscience, China) of paraffin sections in accordance with the manufacturer’s protocol. The number of positive cells was counted using the ImageJ software.

**Statistical analysis**

IBM SPSS Statistics 23 and Graphpad Prism 8 software (Graphpad) were used for statistical data analysis. Categorical variables were compared by Chi square test or Chi square test with Yate’s correction. Student’s two-tailed t test was used for pairwise comparisons. Ordinary one-way ANOVA followed by Tukey’s or Dunnett’s multiple comparisons test was used for multiple comparisons. Data are presented as the mean ± standard error of the mean (SEM), unless otherwise indicated. In all figures and figure legends, “n” represents the number of samples or animals utilized in the indicated experiments. A P value of less than 0.05 was considered significant. ns not significant; *p < 0.05; **p < 0.01; ***p < 0.001; ****p < 0.0001.Selections of the statistical analysis methods meet our data analysis.

**Figures**


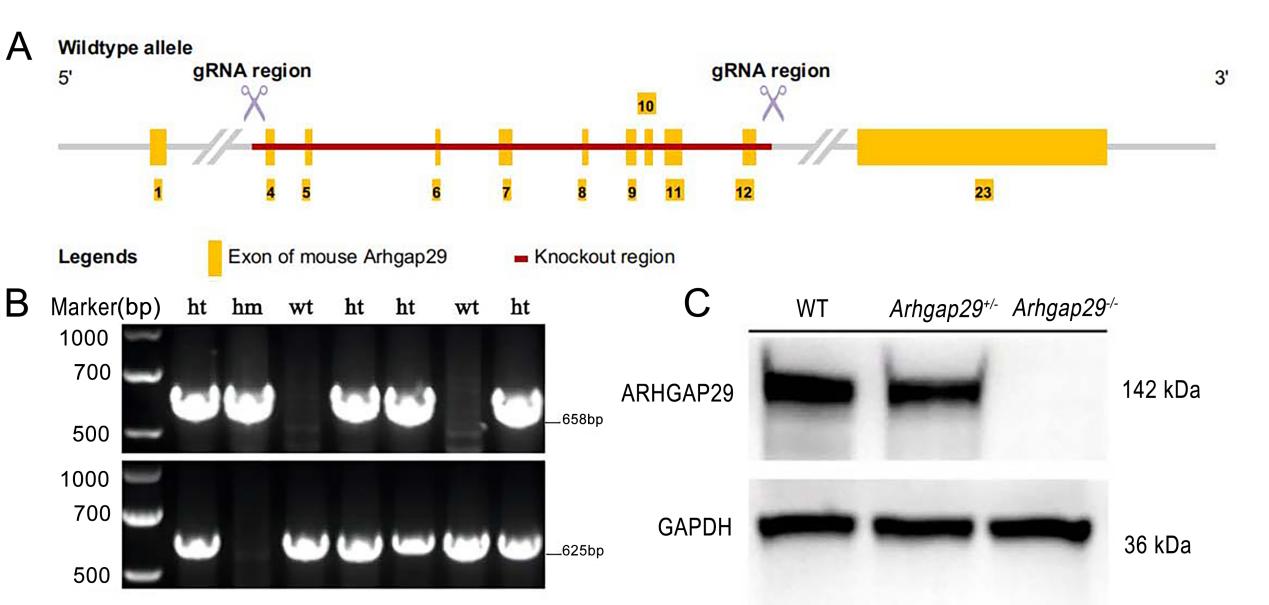


**Figure S1.** Characterization of the *Arhgap29^−/−^* mice. **(A)** *Arhgap29* knockout mouse model (C57BL/6J) by CRISPR/Cas-mediated genome engineering. Twenty-three exons were identified (yellow), with the ATG start codon in exon 2 and the TAG stop codon in exon 23 (Transcript: ENSMUST00000037958). Exons 4–12 were selected as target sites by scissors showing (red). **(B)** Polymerase chain reaction genotyping results. The homozygous (hm) lane shows one band with 658 bp; the heterozygous (ht) lane shows two bands with 658 bp and 625 bp; the wild-type allele lane shows one band with 625 bp. **(C)** Western blot test of ARHGAP29 protein in P0 palatal tissues from the indicated genotypes.


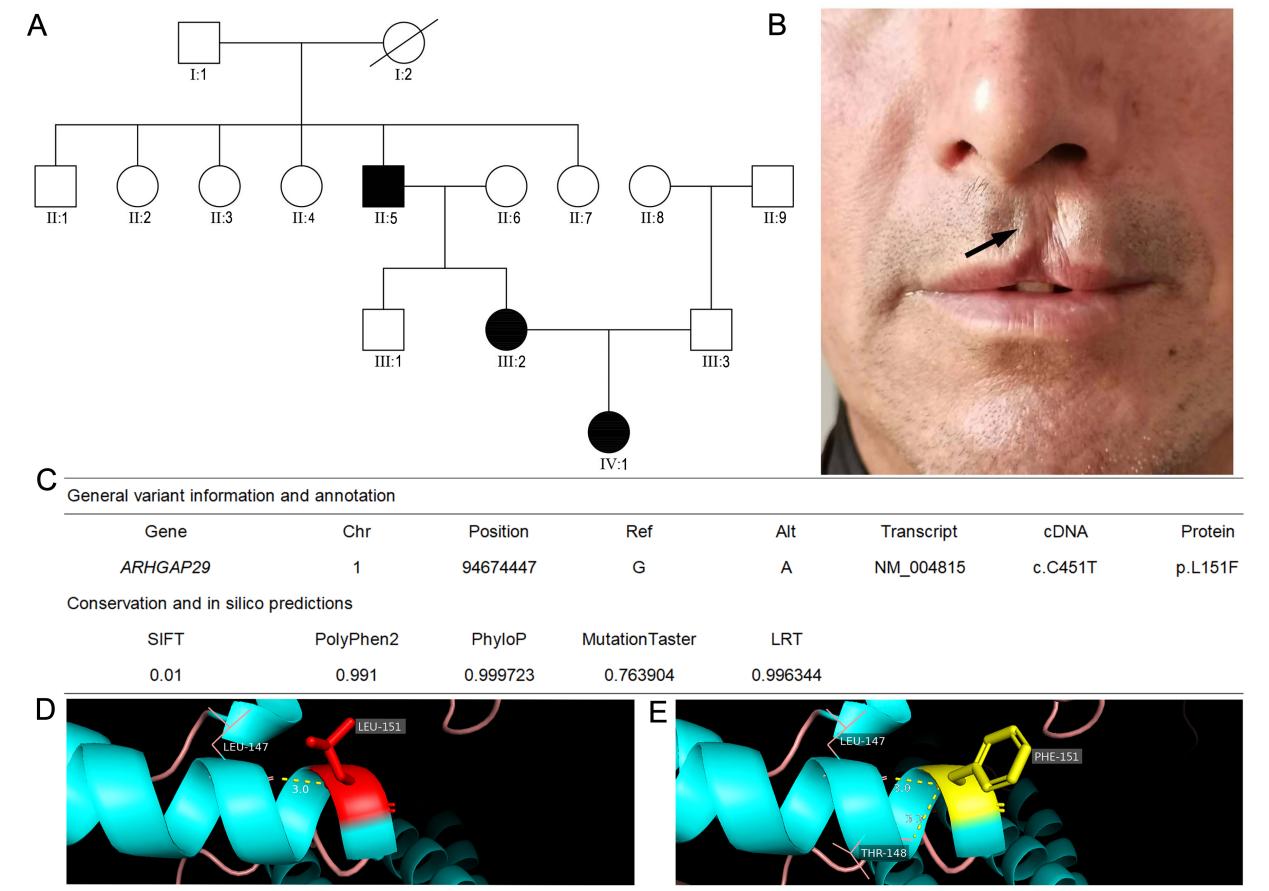


**Figure S2.** Pedigree information and SNP in *ARHGAP29* analysis. **(A)** Pedigree map of the three-generation CL/P family. The affected members (black symbols) include proband (III:2), her father (II:5), and her daughter (IV:1). **(B)** The cleft lip phenotypes of the proband’s father. The black arrow indicates the position of the cleft lip. **(C)** General variant information and annotation. **(D)** The protein structure prediction result of wildtype. **(E)** The protein structure prediction result of the mutation.


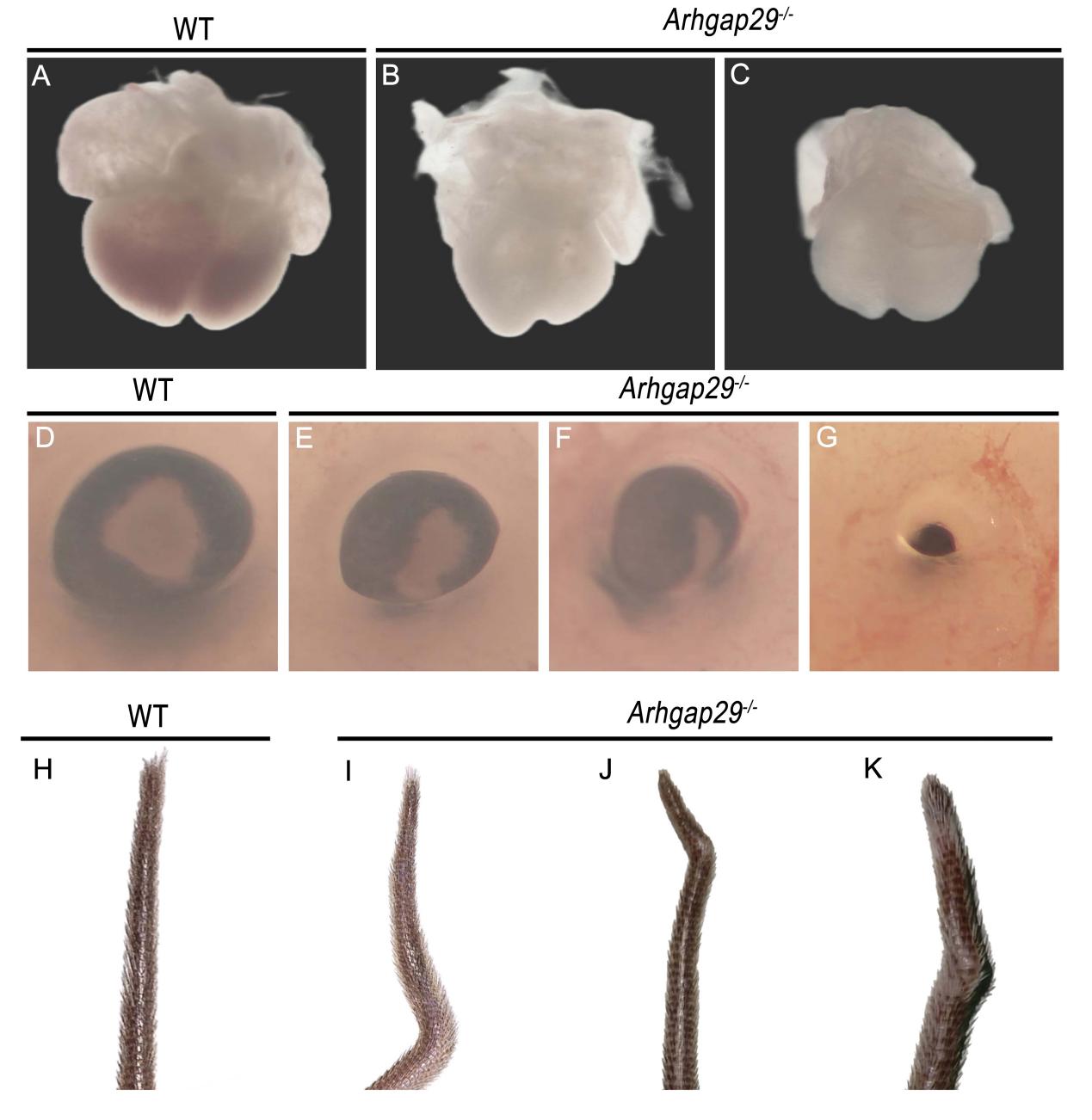


**Figure S3.** *Arhgap29^−/−^* mice exhibited EEC-like multiple malformations at E14.5 and adulthood. Views of hearts obtained from control **(A)** and *Arhgap29^−/−^* mice **(B)** and **(C)** at E14.5. **(D)** Normal eye of wild-type mice. **(E)** Slight microphthalmia with a slightly abnormal pupil shape in *Arhgap29^−/−^* mice at E14.5. **(F)** Moderate microphthalmia with uveal coloboma. **(G)** Severe microphthalmia. The phenotype of the *Arhgap29^−/−^* mice showed kinked tail tips **(I)**, **(J)**, and **(K)** compared with control **(H)** in adulthood.


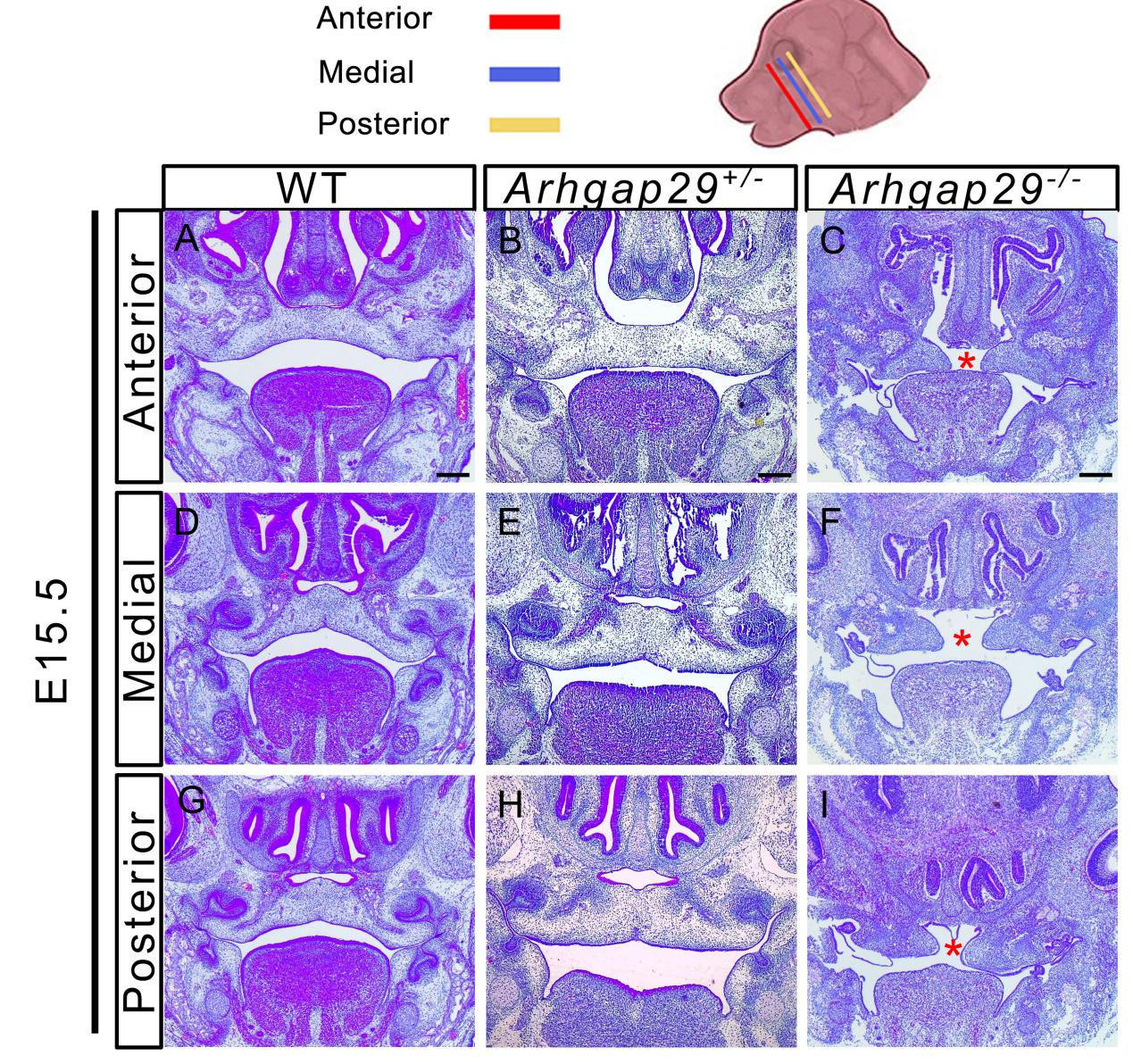


**Figure S4.** Palatal shelves displayed developmental defects in *Arhgap29^−/−^* and *Arhgap29^+/−^* mice compared with wild-type mice at various time points and positions. H&E staining of palatal shelves at different positions (anterior, medial, and posterior palate) compared among different genetic groups at E15.5 **(A–I)**Asterisk: cleft palate; scale bar = 500 μm. Diagrammatic drawing of the mouse head in the top image: position and angle of continuous coronal sections of anterior (red), medial (blue), and posterior (yellow) at E14.5 and E16.5.


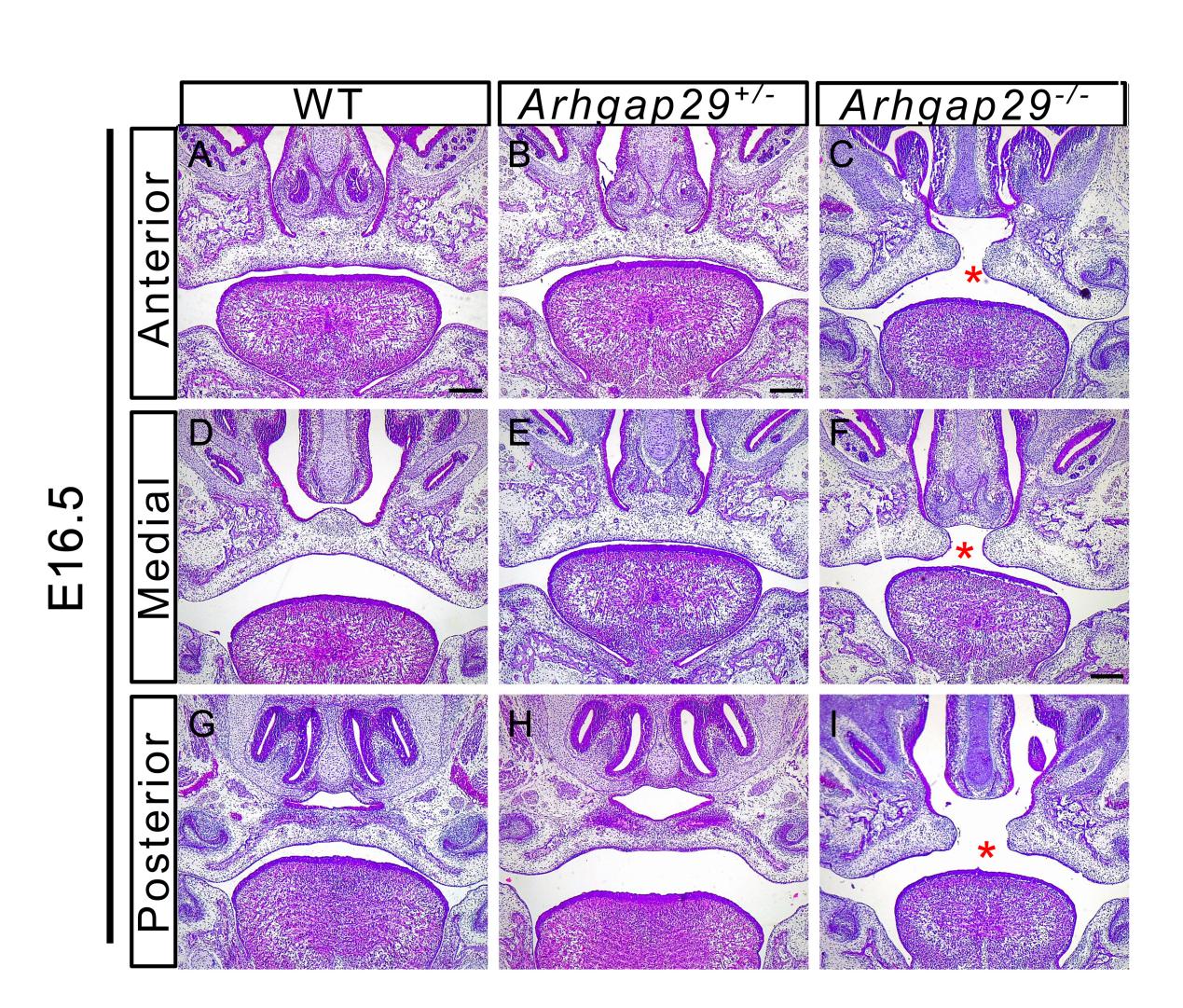


**Figure S5.** Palatal shelves displayed developmental defects in *Arhgap29^−/−^* and *Arhgap29^+/−^* mice compared with wild-type mice at various time points and positions. H&E staining of palatal shelves at different positions (anterior, medial, and posterior palate) compared among different genetic groups at E16.5 **(A–I)**Asterisk: cleft palate; scale bar = 500 μm.


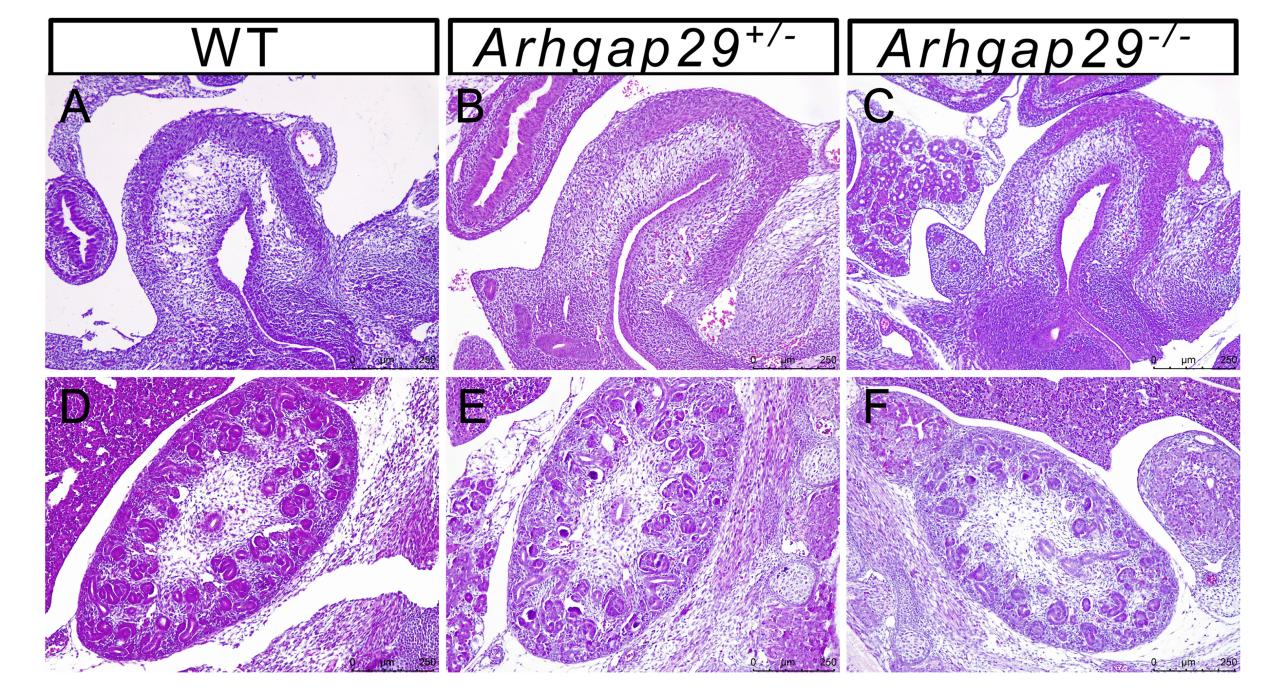


**Figure S6.** H&E staining of continuous coronal sections of bladder and kidney in wild-type mice and mutant embryos. The upper images show the entire bladder of E14.5 wild-type **(A)**, *Arhgap29^+/-^* **(B)**, and *Arhgap29^-/-^* embryo **(C)**. The lower images **(D)**, **(E)**, and **(F)** indicated the entire kidney of wild-type, *Arhgap29^+/-^*, and *Arhgap29^-/-^* embryo, respectively.
